# Adverse local tissue reactions in arthroplasty: opportunities and challenges for a common terminology across scientific, clinical and regulatory fields

**DOI:** 10.1530/EOR-2024-0116

**Published:** 2025-05-05

**Authors:** Anastasia Rakow, Frank Schulze, Janosch Schoon, Ivan De Martino, Giorgio Perino

**Affiliations:** ^1^ Center for Orthopaedics, Trauma Surgery and Rehabilitation Medicine, University Medicine Greifswald, Greifswald, Germany; ^2^ Adult Reconstruction and Joint Replacement Service, Division of Orthopaedics and Traumatology, Fondazione Policlinico Universitario Agostino Gemelli IRCCS, Roma, Italy; ^3^ Università Cattolica del Sacro Cuore, Roma, Italy

**Keywords:** adverse local tissue reactions (ALTRs), arthroplasty implants, implant failure, metal wear debris, adverse event codes, evidence-based lexicon, arthroplasty registries, histopathological analysis

## Abstract

- Clinicians, scientists and regulators do not use a common set of definitions and terminology to classify and code periprosthetic tissue reactions to wear debris of arthroplasty implants and a limited granularity is present to allow early identification of associated adverse events.
- Adverse local tissue reactions (ALTRs) is an umbrella term, which has been used in particular for periprosthetic tissue reactions to metal wear debris. In this review, it has been extended to all implant materials and adverse reaction to metallic debris as a subset of ALTR caused by or associated with metallic particulate debris.
- The high variability in the terminology of ALTRs used by national arthroplasty registries, various coding systems and clinicians impedes their accurate reporting and interpretation, crucial for evaluating the reasons for implant failure and revision arthroplasty.
- Histopathological examination of periprosthetic soft tissue and bone uses standardized criteria for the diagnoses of reactions to wear particles, significantly contributing to their understanding and refining their interdisciplinary terminology.
- This review critically analyzes the current gap in coding ALTRs due to arthroplasty implants’ wear in national registries and classification systems of adverse events and the use of key terms. A comprehensive unified lexicon and classification system grounded on evidence-based histopathological analyses is proposed, implementing the following findings.
  (a) Pseudotumor is a descriptive term for ALTR, which cannot be used for codification.
  (b) Metallosis is a term lacking quantitative and qualitative determination and thus not a codifiable term for ALTR.
  (c) Aseptic lymphocyte dominant vasculitis-associated lesion (ALVAL) should not be used due to absence of histological findings diagnostic of vasculitis.
  (d) Metal delayed hypersensitivity and metal allergy should be codified as separate categories of adverse events.
  (e) ALTR is to be classified in due consideration of definition of predominant lymphocytic or predominant macrophage infiltrate.
  (f) Granulomatous reaction should be reserved to sarcoid-like, immune granulomas separated from the macrophage infiltrate with/without foreign body giant cell reaction.
  (g) Macrophage infiltrate containing particulate wear debris with or without lymphocytic component associated with macrophage induced osteolysis/aseptic loosening should be considered as a type of ALTR.

## Introduction

It has been recently proposed in arthroplasty surgery to shift the focus from standard protocol(s) for every patient toward personalized medicine to match the developments in research based on available data (1). However, the terminology used for the classification of adverse local tissue reactions (ALTRs) to arthroplasty implant wear debris is still taking small, incremental steps to move from terms coined in the past to a standardized, evidence-based comprehensive lexicon (2). This development would be instrumental in the clinical setting, for arthroplasty registries, national data bases and regulatory authorities and for systems collecting data of adverse outcomes related to medical devices, such as the Manufacturer and User Facility Device Experience (MAUDE) database developed by the Food and Drug Administration (FDA), USA. Today, scientists, clinicians and regulators do not necessarily use a common set of definitions and terminology for adverse responses to metal orthopaedic implants across respective fields, which would be preferable to understand them (3).

The term ALTR is preferred in this narrative review to indicate adverse reactions in the periprosthetic soft tissue and bone caused by or associated with any material used in arthroplasty. The term adverse reaction to metallic debris (ARMD) is retained as a subset of ALTRs specifically caused by or associated with metallic wear debris generated by abrasion/adhesion/erosion, third body wear and corrosion modes. The use of a standardized terminology for tribocorrosion-associated ALTRs has been considered essential in a recent publication, although only for some general terms or types of reaction in the periprosthetic soft tissue (4). Yet, much less consideration for the periprosthetic bone is found in the proposed terminology framework, in part because of paucity or lack of bone specimens retrieved at surgery for histopathological examination. The aims of this narrative review article are i) to compare the terminology for ALTRs used in national arthroplasty registries for large joint implants, the International Medical device Regulators Forum (IMDRF) and the International Statistical Classification of Diseases and Related Health Problems (ICD-11) coding system, ii) to discuss the merits and limitations of the histopathological examination in the diagnosis of ALTRs, and iii) to propose codifiable ALTR categories on evidence-based histopathological examination and the classification proposed in a recent review article of the current terminology (5).

This review does not discuss the issue of an ALTRs grading system and the use of histopathological examination for diagnostic purposes already thoroughly examined in the abovementioned article (5).It does not focus on adverse reactions to biomaterials and/or devices used in non-arthroplasty orthopaedics.

## Terminology used in major national arthroplasty registries

Today, registries collect up to four levels of data (6); Level-I data comprise patient, surgeon and hospital identifiers and procedure data, allowing for assessment of revision and reoperation rates, although the definition of implant revision varies significantly among the national arthroplasty registries (7). Level-II data include patient factors, comorbidities, surgical data, perioperative care and complications. Level-III data consist of patient-reported outcome measures, allowing for identification of risk factors for unfavourable outcomes, assessment of postoperative overall health improvement and cost-effectiveness analyses. Level-IV data consist of radiographs, which may help to detect subclinical implant failure, including implant wear and osteolysis.

Revision surgery is considered the primary failure end point in arthroplasty, defined as removal and/or exchange of any implant component, and part of the hierarchical Level I of information (6). However, it is noteworthy that the histopathological examination of the periprosthetic tissue has not been considered in national arthroplasty registries at any hierarchical level of information. In contrast, the examination of implant components was recently proposed at level V of information in a pilot study (6, 8). The ALTR categories for periprosthetic soft tissue and bone used in major arthroplasty registries are presented in Table 1.

**Table 1:** ALTR categories for periprosthetic soft tissue and bone in Major National Arthroplasty Registries. The data were extracted from the most recent national registry report available accessed on June 12, 2024. If the annual report was not available online, the categories were extracted from a recent publication of THA and/or TKA revision data from the respective national registry.

National registry/ implant type	ALTR (periprosthetic soft tissue)	ALTR (periprosthetic bone)
AJRR, USA		
THA/HRA	Infection and inflammatory reaction	Articular bearing surface wear and osteolysis/aseptic loosening
TKA/UKA	Infection and inflammatory reaction, stiffness	Articular bearing surface wear and/or osteolysis, mechanical loosening
AOANJRR, Australia		
THA/HRA	Metal-related pathology, wear of implant component(s)	Aseptic loosening/lysis
TKA/UKA	Metal-related pathology, wear of implant component(s)	Aseptic loosening/lysis
DHR, DKR, Denmark		
THA/HRA	Polyethylene wear wo loosening	Aseptic loosening (one or both components), osteolysis without loosening
TKA/UKA	None	Aseptic loosening
LROI, Netherlands		
THA/HRA	Symptomatic MoM bearing	Loosening of prosthesis acetabular or femoral component
TKA/UKA	None	Loosening of prosthesis component; arthrofibrosis
FAR, Finland		
THA/HRA	Other reasons (please specify)	Aseptic loosening
TKA/UKA	Other reasons (please specify)	Aseptic loosening
EPRD, Germany		
THA/HRA	Wear	Loosening/osteolysis with fixed component(s)
TKA/UKA	Wear, restricted mobility	Loosening/osteolysis with fixed component
NJR, UK		
THA/HRA	Adverse reactions to particulate debris, implant wear	Aseptic loosening/lysis
TKA/UKA	Implant wear, stiffness	Aseptic loosening/lysis
NAR, Norway		
THA/HRA	None, defective polyethylene	Loosening/osteolysis wo loosening of implant component(s)
TKA/UKA	Polyethylene liner wear	Loosening of implant component(s)
SAR, Sweden		
THA/HRA	Adverse event: an unexpected negative event, consequence of joint replacement surgery, for example, an infection	Aseptic loosening: loosening of prosthesis component(s) without proven infection, osteolysis: loss of bone tissue
TKA/UKA	Adverse event: an unexpected negative event, consequence of joint replacement surgery, for example, an infection	Aseptic loosening: loosening of prosthesis component(s) without proven infection, osteolysis: loss of bone tissue

ALTRs, adverse local tissue reactions; AJRR, American Joint Replacement Registry; AOANJRR, Australian Orthopaedic Association National Joint Replacement Registry; DHR, DKR, Danish Arthroplasty Register; EPRD, German Arthroplasty Registry; FAR, Finnish Arthroplasty Register; LROI, Dutch Arthroplasty Register; MoM, metal-on-metal; NAR, Norwegian arthroplasty Register; NJR, National Joint Registry for England, Wales, Northern Ireland, the Isle of Man and Guernsey; SAR, Swedish Arthroplasty Register; THA, total hip arthroplasty; HRA, hip resurfacing arthroplasty; TKA, total knee arthroplasty; UKA, unicompartmental knee arthroplasty.

In a study by the International Consortium for Orthopaedic Registries (ICOR) to elucidate performance of devices in hip and knee arthroplasty, a comparative examination of the categories revealed that the development of a common lexicon for the soft tissue and bone ALTRs has not been considered (9). Yet, considering the rapid rise in the use of artificial intelligence (AI) applications in healthcare, and specifically in arthroplasty with a steady growth of publications, a standardized terminology is key to facilitate effective communication and increase efficiency of the dialogue (10, 11). Still, the categories of ALTRs used in these studies do not reflect these dynamic changes. Current literature lacks any discussion on how to address this important issue regarding scientifically sound reporting of arthroplasty implant performance, thus improving data comparability and, ultimately, patient safety. The adoption of terminology standards to enable exchanges between data information ecosystems has been considered crucial for the improvement of data collection efficiency and the advancement of real-world evidence for medical devices (12).

It is beyond the scope of this review to examine the differences in the lexicon among the registries in detail. However, highlighting the categories of implant failures used by the American Joint Replacement Registry (AJRR) can be a fitting example of the problems affecting the classification of ALTRs. The AJRR does not differentiate between infection of and inflammatory reaction in periprosthetic soft tissue and articular bearing surface wear and osteolysis/aseptic implant loosening in total hip arthroplasty (THA) and total knee arthroplasty (TKA) implants. In addition, this classification has been chosen without further specification or explanation of how the categories have been considered appropriate for capturing the biological differences among ALTRs or be effective for the safety signal detection as a tool to promptly identify risks associated with the use of prosthetic devices (13).

## Terminology suggested by the IMDRF

A coding dictionary of three hierarchical levels to describe adverse events for medical devices has been developed by the IMDRF. Some of the challenges associated with the reporting have been addressed by IMDRF linking their codes to the Medical Dictionary for Regulatory Activities (MedDRA) coding system of five hierarchical levels (14). IMDRF has also developed an adverse event terminology maintenance plan aimed at adding, modifying or deleting adverse event terms of the coding dictionary (15). Despite the attempts to standardize and harmonize the coding system, the development of a more granular level of coding has been recommended to increase specificity when appropriate. This process is considered crucial to enhance the current adverse event coding for medical devices (13). The ALTR categories for periprosthetic soft tissue and bone used by IMDRF with MedDRA link are presented in Table 2.

**Table 2:** ALTR categories for periprosthetic soft tissue and bone in IMDRF and non-IMDRF code.

Level 1 term	Level 2 term	Code	Definition	Non-IMDRF code
MSKS	Metal-related pathology	E1618	Also known as metallosis. Includes trunnionosis, aseptic fibrosis or local necrosis secondary to metal corrosion and release of wear debris	MedDRA:10081986: periprosthetic metallosis
	Osteolysis	E1627	Dissolution of bone; applied especially to the removal or loss of the calcium of bone	MedDRA:1003124: osteolysis
	Osteopenia/osteoporosis	E1629	Decreased calcification or density of bone tissue	MedDRA:1004908: osteopenia
	Synovitis	E1632	Inflammation of a synovial membrane	MedDRA:10042868: synovitis
	Bursitis	E1637	Inflammation of the fluid-filled pad (bursa)	MedDRA:1000681: bursitis
Investigations and diagnostic tests	High metal ion levels	E2206	High blood or serum levels of ions which are attributed to one or more medical devices used, such as but not limited to Co, Cr, Ti, Ni and Mo	MedDRA:1008605: heavy metal increased

ALTRs, adverse local tissue reactions; IMDRF, International Medical Device Regulators Forum; MedDRA, Medical Dictionary for Regulatory Activities; MSKS, musculoskeletal system.

The only category of adverse reaction for the periprosthetic soft tissue is found under ‘metal related pathology’ (code E1618), encompassing metallosis, aseptic fibrosis secondary to metal corrosion, i.e., trunnionosis, and release of wear debris. The definition of the items in this category is problematic because ‘metallosis’ and ‘trunnionosis’ are no medical diagnostic terms, although both have been widely used in literature by orthopaedic surgeons to define the presence of metallic wear debris with a wide range of biological effects. Aseptic fibrosis in contrast to arthrofibrosis is a common neo–synovial reaction not limited to corrosion metallic particles, but also observed with abrasion-/ adhesion-/ erosion-induced metallic and ceramic particle debris and is not considered adverse by definition.

More troublesome are the definitions of osteolysis (code E1627) as a ‘dissolution of bone; applied especially to the removal or loss of the calcium of bone’ and those of osteopenia/osteoporosis (code E1629) as ‘decreased calcification or density of bone tissue’, which confuse the medical definition of osteomalacia with osteolysis, osteopenia and osteoporosis. In a deficit of bone architecture (osteolysis, osteopenia and osteoporosis), the bone mass decreases, but the ratio of bone mineral to bone matrix is normal, whereas it is in osteomalacia that the ratio of bone mineral to bone matrix is low. Moreover, without histopathological examination of undecalcified bone histomorphometry, it would be impossible to assess microarchitecture of bone, bone cellular activity, bone mineralisation and bone remodeling. A fitting case of adverse event requiring histomorphometry examination of bone has been the failure of the Inter-Op® acetabular component (Sulzer Orthopedics Inc., Switzerland) (16, 17). At last, the category of high metal ion levels (code E2206) seems rather undefined since it lacks a threshold value to define the significance of ‘high’ and does not specify if this value needs to be obtained in whole blood, serum, synovial fluid or other body fluids or tissues. These errors and/or lack of precision for the definition of the codes are problematic and limit (if not prohibit) the application of this coding dictionary as the gold standard employed by a consortium of regulatory agencies worldwide.

## Terminology used in the ICD coding system

The ICD-11 coding system, a three-part model for coding causes and mechanisms of healthcare-related adverse events has been considered a ‘promising new way to capture healthcare-related harm or injury’ (18). The codes provided for ALTRs in the periprosthetic soft tissue and bones are presented in Table 3.

**Table 3:** ALTR categories for periprosthetic soft tissue and bone in the ICD-11 coding system.

ICD-11 code	Definition
FA35.Z	Wear of articular bearing surface of joint prosthesis of unspecified joint
FA35.0	Wear of articular bearing surface of joint prosthesis of hip
FA35.1	Wear of articular bearing surface of joint prosthesis of knee
FA35.2	Wear of articular bearing surface of joint prosthesis of other joint
FC01.8	Postsurgical osteolysis
NE83	Injury or harm arising from other device, implant or graft, not elsewhere classified
NE83.1	Infection and inflammatory reaction due to internal joint prosthesis

ALTRs, adverse local tissue reactions; ICD-11, 11th version of the International Statistical Classification of Diseases and Related Health Problems of the World Health Organization.

The code of ‘wear of articular bearing surface of joint prosthesis’ (FA35) in this definition does not provide any indication of an adverse event since a certain amount of wear is physiologically expected. In similar fashion, the code for ‘post-surgical osteolysis’ (FC01.8) does not qualify for an adverse event without any specified quantification of the process or the addition of loosening of implant components. In fact, periprosthetic osteolysis per se always occurs postsurgically to some extent. In addition, the code for ‘infection and inflammatory reaction due to internal joint prosthesis’ (NE83.1) unifies infectious and delayed hypersensitivity/allergy complications under the same code rendering the report of both, incidence and prevalence, impossible. This terminology has been adopted by the AJRR for periprosthetic soft tissue adverse reaction.

## Terminology derived from histopathological classification

In 2014, a comprehensive, revised histopathological classification for periprosthetic soft tissue and bone reactions has been proposed based on tens of thousands of observations (19).

The histopathological classification for the periprosthetic soft tissue can be extended to the involvement of bursal synovium, which has occurred with some frequency in cases of corrosion-associated wear, causing disruption and necrosis of the surrounding tissues (5). This classification can be complemented by the specification of the type of wear particles observed in the periprosthetic tissue (20). The inclusion of these histopathological criteria in terminology frameworks used by governing bodies of the national arthroplasty registries and the regulatory agencies has the potential to provide a more accurate classification of ALTRs, keeping pace with scientific and technological progress.

Another remarkable and unexplained aspect of reporting the reasons for revisions of arthroplasty implants is the wide discrepancy between the terminology used by the coding systems and the one adopted following evidence-based observations in the clinical setting and object of hundreds of publications. Some of the most common terms used are often mistaken as or confused with histopathological diagnoses. These are critically discussed below to provide a frame of their correct use in the classification of ALTRs as reasons of implant failures or their deletion from the current lexicon.

### Pseudotumor

The term pseudotumor, although considered of practical use for clinical and radiological diagnosis of ALTRs (21), is not a codifiable diagnostic entity by histopathological examination, and the lymphocytic predominant ALTR originally described as aseptic lymphocyte dominant vasculitis-associated lesion (ALVAL) represents only a distinctive subset (22).

Above all, pseudotumor cannot be used and/or validated as a cause for revision or failure of arthroplasty implants in any coding system for the following reasons: i) there is no threshold value for its definition regarding size, volume and neo-synovium thickness; ii) its natural history and clinical outcome vary according to cell composition and anatomical site; iii) extracapsular extension varies according to the cell composition and fluid accumulation of its intracapsular component; iv) the growth rate of the macrophage predominant type depends on wear particle rate and physicochemical properties and host factors; v) the macrophage predominant type is nonspecific and can occur with accumulation of wear particles of any material in the macrophages associated with a variable degree of micropapillary to polypoid fibrovascular proliferation, with slow increase in size during implantation time in well-functioning implants; and vi) organized hematomas from chronic hemorrhage or large seromas can be radiologically misdiagnosed as ALTRs with lymphocytic predominant pattern (5). Examples of intracapsular and extracapsular pseudotumors and their natural history are well-illustrated in a recent review article on the histopathology of ALTRs (5).

### Metallosis and ALVAL

Like the term pseudotumor, metallosis cannot be considered a codifiable diagnostic entity, because it is defined by the presence of a variable amount of abrasion-, adhesion-, erosion-, tribocorrosion- or corrosion-associated wear products in the joint fluid, periprosthetic soft tissue and/or bone marrow without a threshold value of reference. This holds particularly true for metal-on-metal (MoM) hip resurfacing arthroplasty (HRA) and THA implants, which can generate only metal-derived wear particles, ions, oxides and salts (23). Although popular for decades in the orthopaedic clinical community and used in many publications, the term metallosis should be substituted by the presence of these metal-derived degradation products as for all the other implant materials, with specification of how wear is generated (abrasion/adhesion/erosion/tribocorrosion/corrosion or mixed) when appropriate. Correlation with type and degree of inflammatory infiltrate in the periprosthetic tissue with state-of-the-art analytical techniques should be considered (24, 25).

The lymphocytic dominated type of ARMD, either associated or not with a variable degree of soft tissue/bone necrosis, was originally described as ALVAL in 2005 (22). However, the evidence of vasculitis was not found in subsequent histopathological analyses, with the most consistent finding being the presence of high endothelial cell venules (5). Therefore, the term ALVAL should not be used to define the lymphocyte-predominant type of ARMD with or without soft tissue transmural necrosis.

### Granuloma

Two broad forms of well-defined tissue reactions with presence of a variable number of multinucleated giant cells have been histologically documented, defined by their etiology: the so-called foreign-body giant cell (FBGC) reaction and the immune granuloma (26). FBGCs are formed only in presence of wear particles or agglomerates/aggregates of wear particles that exceed the phagocytic capacity of single cells, thus resulting in FBGC reaction without an adaptive immune response and in immune granulomas with response to an immunogenic stimulus of the adaptive immune system (26, 27). In cases of particulate wear debris from any material, the term granulomatous reaction should be reserved to the immune granuloma, characterized by the formation of distinct aggregates of epithelioid macrophages, with scattered multinucleated giant cells separated by a variable amount of stroma (sarcoid-like) with or without the presence of a lymphocytic cuff often accompanied by plasma cells. It has shown the presence of a mixture of M1/M2 macrophages and Th1 and Th2 lymphocytes (28), suggesting the possibility of a hypersensitivity reaction type IVa and possible activation of metal particle specific CD4+ T lymphocytes through migration of dendritic cells to regional lymph nodes, as in beryllium-induced hypersensitivity (29).

Therefore, it is proposed that the granulomatous ALTR is reserved to the immune granuloma, while the FBGC reaction considered to be within the physiological range of responses to wear debris, unless associated to macrophage induced osteolysis. However, the histological differential diagnosis between FBGC reaction and immune granuloma may be complicated by the possible evolution from one type to the other.

**Figure 1:**
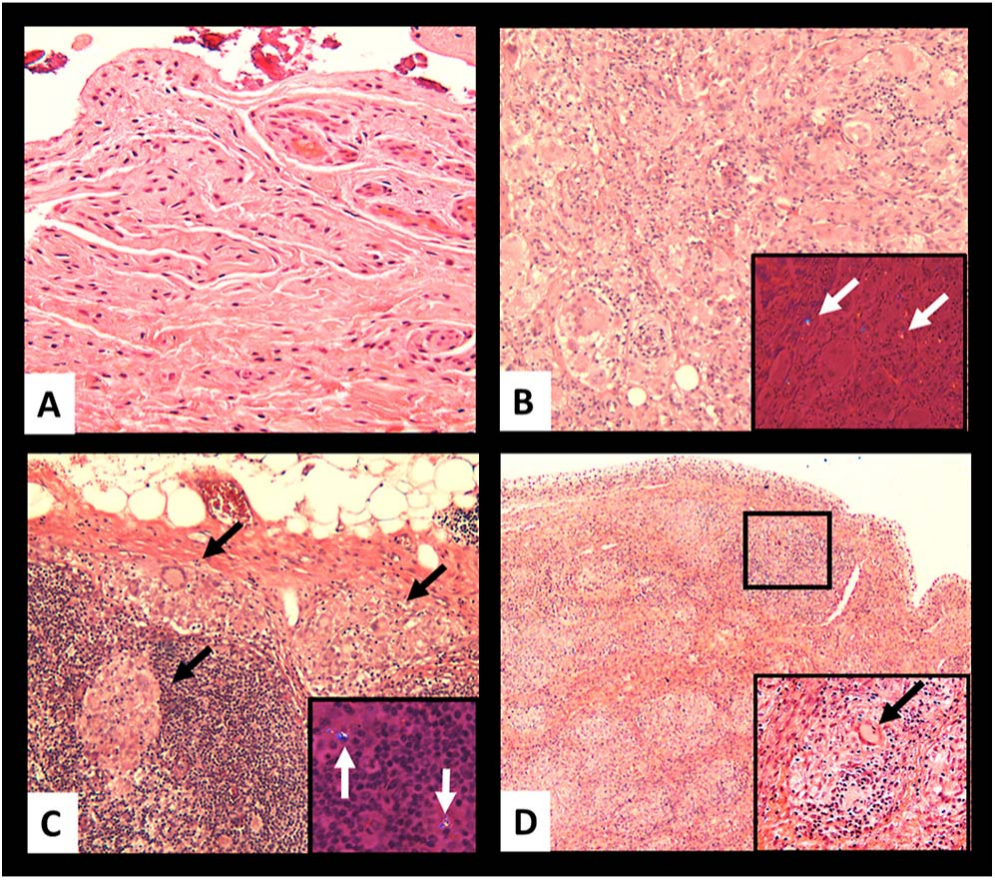
Sarcoid-like granulomatous reaction to polyethylene wear debris. (A) Fibrotic synovium, patellofemoral region, time 0; (B) florid macrophage reaction with multinucleated foreign body giant cells and admixed lymphocytes to PE debris is evident in inset under compensated polarized light, TOI: 17 months (first UKA implant revision). (C) Regional lymph node with formation of non-necrotizing granulomas in subcapsular and cortical location (black arrows) containing PE debris in inset (white arrows), TOI: 22 months; (D) progression to sarcoid-like granulomatous reaction with Touton multinucleated giant cell in inset (black arrow), TOI: 24 months (second UKA implant revision with conversion to TKA). PE, polyethylene; TOI, time of implantation; UKA, unicompartmental knee arthroplasty; TKA, total knee arthroplasty.

**Figure 2:**
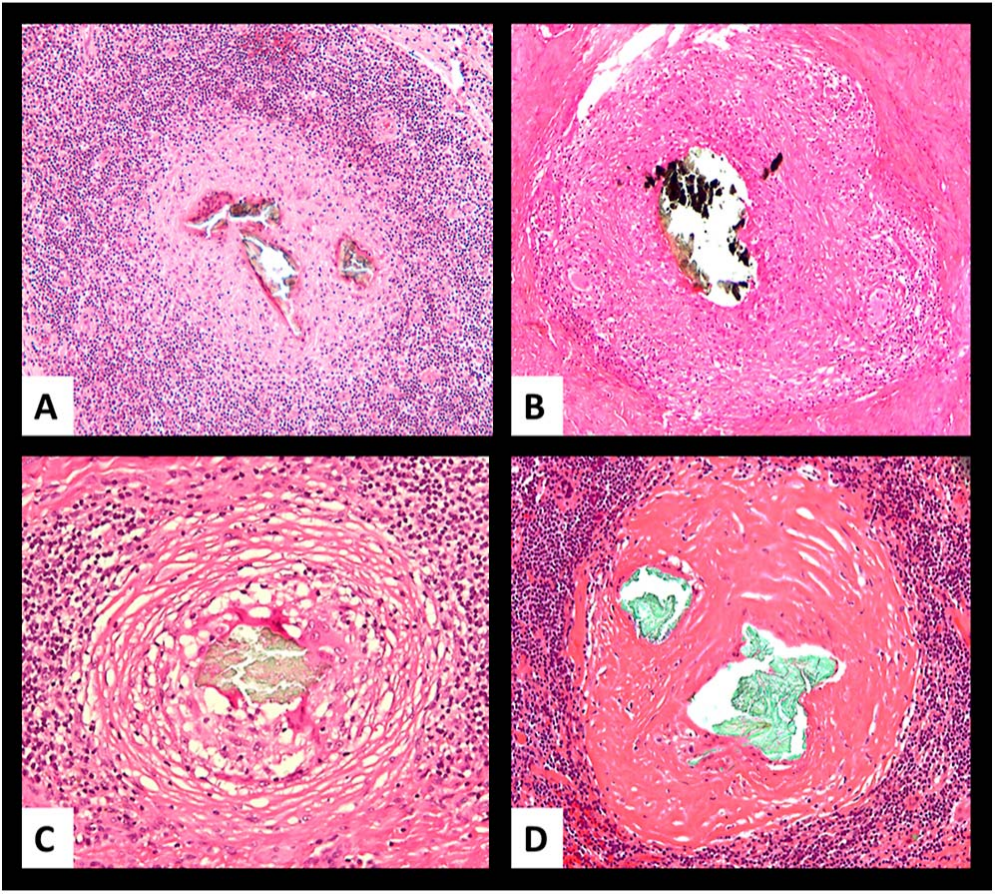
Sarcoid-like granulomatous reaction to corrosion of metal wear debris. (A) Epithelioid cell granuloma with occasional foreign body multinucleated giant cells and without lymphocytic cuffing surrounding a central aggregate of metallic wear nanoparticles in case of MoM THA; (B) epithelioid cell granuloma with occasional foreign body multinucleated giant cells and lymphocytic cuffing with scattered plasma cells (cognate granuloma) surrounding a central aggregate of metal wear nanoparticles in case of MoM THA; (C) epithelioid cell granuloma with lymphocytic cuffing showing a moderate degree of fibrosis and surrounding a central aggregate of metal wear nanoparticles in case of non-MoM THA with CoCr DMN; (D) end stage fibrotic granuloma with lymphocytic cuffing and diffuse hyalinisation and surrounding a central aggregate of metallic wear nanoparticles in case of MoM THA. MoM, metal-on-metal; THA, total hip arthroplasty; DMN, dual modular neck.

The first example is provided in a case of reaction to polyethylene (PE) debris, sharing features with a case previously published in the literature (30) and illustrated in Fig. 1A, B, C, D, showing progression from florid FBGC reaction to immune, sarcoid-like granulomas. The second example is provided by the natural history of immune granulomas in cases of agglomerates/aggregates of nanoparticles from metal wear debris generated by various corrosion modalities and presented in Fig. 2A, B, C, D, showing the evolution of the immune granuloma from active to fibrotic nodules with hyalinisation. This transition might reflect a diminished biological activity of the nanoparticle aggregates after embedding into the periprosthetic neo-synovium due to properties acquired during their interaction with the synovial fluid, ultimately leading to a loss of immunogenicity.

### Osteolysis and aseptic loosening

The occurrence of osteolysis and consequently aseptic loosening is considered an adverse event in the periprosthetic bone by the national arthroplasty registries and regulatory agencies; however, further specification is not provided in any of the classifications.

The vast literature published on experimental and clinical osteolysis as the main cause of aseptic loosening has been summarized in several comprehensive review articles (31, 32, 33, 34, 35, 36). However, aseptic loosening is the end stage of a process that originated through different mechanisms involving mechanical and/or biological factors. It would be important to separate the classification of osteolysis due to stress shielding of bone from osteolysis as a secondary reaction driven by macrophages containing wear particles to attain conceptual clarity and proper classification and coding.

The report of adverse events in total ankle arthroplasty (TAA) offers a fitting case study to illustrate the importance of osteolysis/aseptic implant loosening subclassification. An evidence-based classification system of complications of TAA has been proposed, including aseptic loosening without further specification (37); however, inconsistency in the reporting of adverse events secondary to lack of consensus guidelines or a validated classification system has been reported with the term loosening/osteolysis listed in 80/117 (68.4%) studies analyzed (38). An attempt of subgrouping aseptic loosening has been provided by the FDAs MAUDE voluntary database, which includes i) severe osteolysis, ii) loose implant and iii) subsidence (39). Three histopathological studies of periprosthetic osteolysis in TAA have consistently shown macrophage infiltrate in the bone marrow, with FBGC containing birefringent material under polarized light, consistent with PE debris (40, 41, 42).

Limited availability of clinical specimens is the main reason why macrophage and/or wear particle-induced osteolysis has been almost exclusively studied with surrogate experimental animal models, such as the murine calvarium. The study of human samples has been almost invariably limited to the analysis of periprosthetic soft tissue without matching observations of the periprosthetic bone. Consequently, assumptions have been made on the natural history of osteolysis and the molecular pathways in the bone microenvironment without corroboration of the histopathological examination of bone samples *in vivo*.

**Figure 3:**
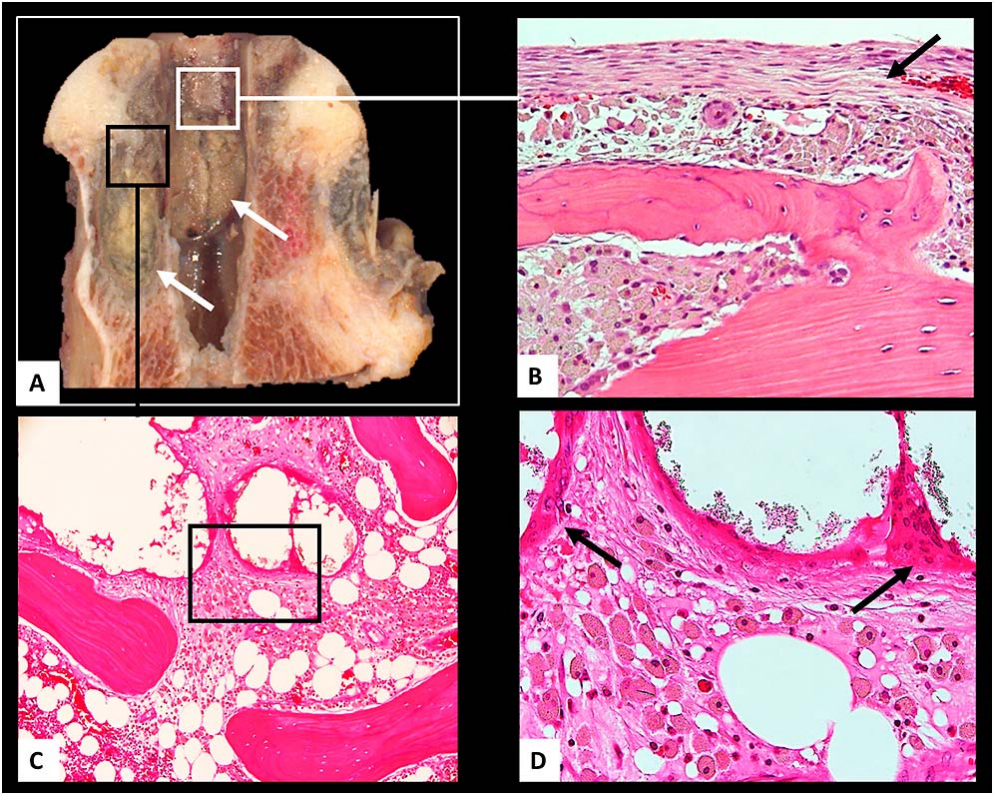
Osteolysis. (A) Femoral head with large areas of osteolysis (white arrows) in a case of MoM HRA, TOI: 49 months, blood serum Co 70 μg/L; Cr 71 μg/L; (B) area in white box showing interposition of a macrophage layer between a fibrous layer adjacent to the metal implant stem (black arrow) and a layer of remodeled periprosthetic bone; (C) area in black box of A showing infiltration of bone marrow by PMMA bone cement and macrophage infiltrate; (D) area in black box of B showing inert giant cell reaction to PMMA bone cement (black arrows) and diffuse particle laden macrophage infiltrate with infiltration of fatty marrow and interstitial space between areas of PMMA bone cement indicative of macrophage motility. MoM, metal-on-metal; TOI, time of implantation; HRA, hip resurfacing arthroplasty; PMMA, polymethyl methacrylate.

**Figure 4:**
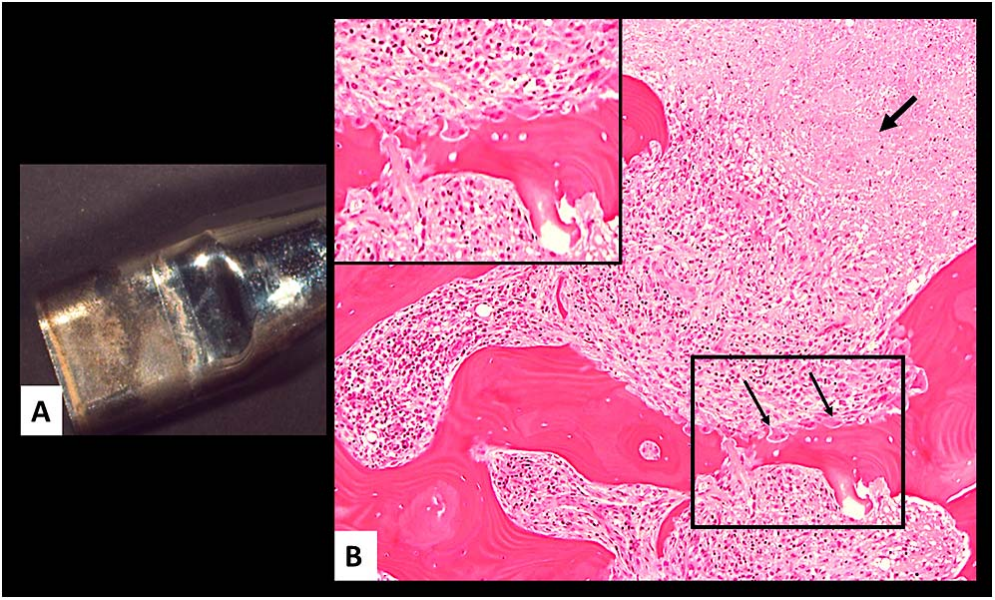
Osteolysis of cortical bone of proximal femur in a non-MoM THA with CoCrMo DMN and TMZF stem, TOI: 12 months, blood Co 20 μg/L, Cr 3 μg/L. (A) DMN showing metal corrosion of the neck/stem male side. (B) Mixed macrophage/lymphocytic infiltrate pushing border with a front of marked osteoclastic activity (black arrows), with high magnification of black box in inset, and area of necrotic tissue (thick black arrow). DMN, dual modular neck; MoM, metal-on-metal; THA, total hip arthroplasty; TOI, time of implantation; TMZF, titanium-molybdenum-zirconium-iron; TOI, time of implantation.

The histopathological examination of periprosthetic bone samples has indicated that the macrophage infiltrate in the periprosthetic bone marrow is the major contributor to the onset and progression of macrophage induced osteolysis, as shown in Figs 3 and 4. The histological findings are corroborated by the following observations.

### Macrophage motility and migration from neo-synovium to bone marrow

This evidence-based reconstruction of the osteolytic process secondary to metal debris generated by various forms of corrosion can be extended to the toxicity and/or immunogenicity of PE debris dependent on the size, shape and especially state of oxidation of the particles (43). Macrophage infiltrate with PE debris has been reported since the late 1970s in cases of acetabular cup osteolysis (44, 45). Macrophage ameboid and mesenchymal motility has been found dependent on the architecture of the matrix (46, 47), with plastic response to metal ions and nanoparticles (48). Wear particle analyses suggest that the presence of Co-rich particles is associated with macrophage migration from the neo-synovium and within the bone marrow (24, 49). These observations are in contrast with the hypothesis that a downstream effect of Co- but not Cr-induced reactive oxygen species (ROS) leads to inhibition of migration (50).

### Periprosthetic soft tissue environment / bone marrow environment

Recent advancements in mechanobiology have shown dependence of macrophage activity on the extracellular matrix structure (51), which can vary between neo-synovium and fatty marrow, especially in regard to the prevalent cytokines and chemokines. The hypothesis of related immunogenicity of the bone marrow as an organ also deserves proper attention (52).

### Correlation between wear particle oxidation and macrophage motility

The difference in the oxidative state between PE/metal-derived particles (53) and ceramic particles of third and fourth generation might provide an explanation why the latter wear debris exhibits a predominant fibrous reaction in the periprosthetic soft tissue and very limited occurrence or absence of aseptic loosening/osteolysis is reported in series of ceramic-on-ceramic (CoC) THA implants (54, 55, 56). The only exception is a well-documented series of 103 CoC THA, with follow-up range between 60 and 125 months reporting 12 cases with linear osteolysis and eleven cases with scalloping expansile-type osteolysis and histological examination of bone specimens of 13 cases, of which four by transmission electron microscopy (57).

### Macrophage and osteoclast activation

The macrophage infiltrate in the bone marrow appears to advance with a pushing border and the marked increase in the osteoclastic activity representing an adjuvant mechanism. The presence of macrophages possibly downregulates levels of osteoprotegerin (OPG) associated to the local expression of receptor activator of NF-kB ligand (RANKL) and ROS formation, thereby upregulating the differentiation of osteoclasts and bone resorption (34) (Fig. 4B). The possible adverse effects of macrophage motility and accumulation in other tissues (58, 59) have not yet been determined and may represent a concern at long-term exposure, especially in the younger population.

### Frustrated phagocytosis reaction to PMMA bone cement and radiographic contrast material

The histopathological examination has shown that the flattened foreign body giant cell around large aggregates of poly(methyl methacrylate) (PMMA) bone cement containing particles of radiographic contrast material is an example of frustrated phagocytosis and does not contribute to osteolysis (Fig. 3C and D).

In conclusion, evidence-based histopathological analysis shows that macrophage-induced osteolysis should be identified as a distinctive cause of implant loosening with wear particle specification and correlation with radiological studies and implant retrieval analysis.

## The value of histopathological examination for terminology definition

Conventional histopathological examination of periprosthetic soft tissue and bone should be mandatory since it significantly contributes to definitions and diagnoses of tissue reactions to implant- and non-implant-related particulate material and to the improvement of interdisciplinary understanding of biological mechanisms of the reactions. Its values and limitations are summarized in Table 4.

**Table 4:** Merits and limitations of the histopathological examination.

Merits	Limitations
Analysis of cellular composition of ALTRs with classification of subtypes with adjuvant techniques	Amount of information provided by light microscopy examination with H&E staining
Identification and reporting of ‘sentinel’ cases of ALTRs to national arthroplasty registries and regulatory authorities	No predictive value for long-term outcome and systemic effects
Clarifying the natural history of ALTRs through standardized tissue sampling and pathology registry as part of the international consortium of national arthroplasty registries	Need of an invasive procedure (fine-needle biopsy or revision/reoperation surgery)
Intracellular particle analysis (soft tissue and bone) with state-of-the-art technology when appropriate	
Repository of periprosthetic tissue for re-testing with development of new technologies	
Histological slide scanning for case sharing, education and AI generated data analysis	
Identification of lines of clinical, basic and translational research	

AI, artificial intelligence; ALTRs, adverse local tissue reactions; H&E, hematoxylin and eosin.

## Radiological-histopathological correlation

**Figure 5:**
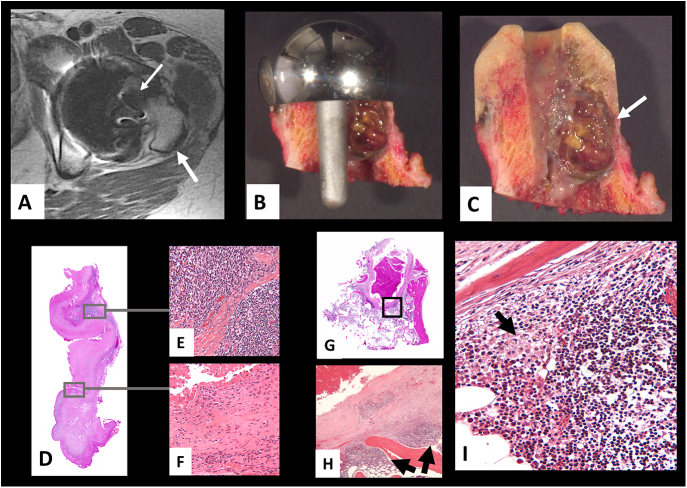
Radiological-histopathological correlation in a case of MoM HRA. (A) MAVRIC-MRI image showing intracapsular accumulation of fluid (high signal intensity) with variable thickness of the pseudocapsule (thick white arrow) and area of osteolysis (low signal intensity) in the femoral head/neck junction (thin white arrow); (B) metal stemmed femoral cup inserted in the proximal femur to resurface the femoral head showing areas of osteolysis; (C) femoral head/neck stump with orthopedic cement cap showing full extension of the osteolytic cavity (white arrow); (D) histological section of the articular pseudocapsule; (E) dense lymphocytic aggregates; (F) neo–synovium fibrosis with loss of surface layer; (G) histological section of area of osteolysis with cavity filled with necrotic macrophage cell debris; (H) area in black box of G showing lymphocytic aggregates and wall of necrotic area lined by particle laden macrophages; (I) high power of lymphocytic aggregate admixed with particle laden macrophages (black arrow). MoM, metal-on-metal; HRA, hip resurfacing arthroplasty; MAVRIC-MRI, multiacquisition variable-resonance image combination-MRI.

Evidence of positive radiological-histopathological correlation is important because reporting of ALTRs is based in most of cases on radiological findings without histopathological analysis confirmation, although radiological examination cannot completely substitute histopathological examination in cases of complex differential diagnosis of associated clinical conditions mimicking an adverse reaction to wear particles and metal ions. Good correlation between metal artifact reduction sequence (MARS) or multi-acquisition with variable resonance image combination (MAVRIC) and histopathological analysis with tissue sampling at surgery with magnetic resonance imaging (MRI) guidance has been reported for MoM HRA and MoM THA (19, 20, 60, 61, 62), as illustrated in a case of MoM HRA in Fig. 5.

In a recent review article, MRI has been found to be a better imaging modality for longitudinal surveillance of ALTRs in MoM HRA and THA (63) and the use of MARS/MAVRIC MRI has been crucial in the identification of cases confirmed by histopathological analysis to be lymphocytic, predominant at early or late stage of development with soft tissue/bone necrosis and extracapsular involvement. MRI is also the best modality to assess macrophage induced osteolysis, with measurement of the volume of the neo-synovium proliferation in high-risk patients through image processing-based methods (64). However, a histopathological study published in 2016 found that the trend of ALTRs, for MoM HRA and THA after the recall of the articular surface replacement (ASR^TM^), THA and HRA system, was the predominant macrophage type with limited amount of fluid and undetermined risk of periprosthetic osteolysis (65). Recent reports have confirmed the finding, showing a steady decline in the revision burden of MoM hip arthroplasties (66), even in ASR^TM^ cohorts (67).

A summary of the terminology for ALTRs involving the periprosthetic soft tissue and bone based on histopathological and radiological findings, considering the histological subtypes of the adverse reaction to corrosion and metal wear debris (65) and the two major types of aseptic osteolysis (68), is presented in Table 5.

**Table 5:** Proposed ALTR categories for periprosthetic soft tissue and bone in evidence-based histopathological and radiological examination.

ALTR categories	
Periprosthetic soft tissue	Periprosthetic bone
Immunological reaction to particulate metal debris with histopathological evidence of predominant lymphocytic component (>grade 2) w/wo soft tissue necrosis and w/wo extracapsular extension (bursitis) or radiological (MRI) findings consistent with these criteria	Mechanical osteolysis (micromotion at the interface surface, stress shielding and others) with implant micromotion / loosening
Allergy reaction to implant material with confirmatory cutaneous hypersensitivity test and histopathological examination of periprosthetic tissue with lymphocytic inflammatory infiltrate	Macrophage/particle wear-driven osteolysis (PE, metal, PMMA cement, ceramic, mixed and others) w/wo implant micromotion/loosening or radiological (MRI) findings consistent with these criteria
Wear particle reaction of predominant macrophage component w/wo FBGC associated with extracapsular extension (bursitis) and/or macrophage/particle wear driven osteolysis with/without implant loosening	Osteonecrosis
Granulomatous type (immune/sarcoid-like granulomas)	
Arthrofibrosis	

ALTRs, adverse local tissue reactions; FBGC, foreign body giant cells; MRI, magnetic resonance imaging; PE, polyethylene; PMMA, poly (methyl)methacrylate; w/wo, with/without.

The issue of the threshold value for ALTR of the macrophage predominant pattern by semiquantitative analysis and evaluation of cell necrosis cannot be resolved by conventional histopathological examination and needs data provided by more sophisticated analytical techniques. The proposed classification of ALTRs based on real-world medical evidence is only a first, necessary step for the formulation of a glossary. The interpretation of the corresponding lemmas validated by a multidisciplinary team of experts (clinicians, basic researchers, regulators and industry scientists) may lead to the construction of a complete domain terminology and a domain ontology in cooperation with experts of information technology (69).

## Conclusion

A common coding dictionary for ALTRs with clear definition for each entity should be developed for national arthroplasty registries, regulatory agencies and the ICD system based on the classification of arthroplasty implant-related pathology. Periprosthetic tissue sampling at revision surgery for histopathological analysis should become a mandatory, standard procedure with pseudo-/anonymized pathology findings using coherent terminology submitted to the national joint registries, and when appropriate, to regulatory agencies. To attain terminological precision and thereby prevent misdiagnoses, ALTR is to be classified in due consideration of definition of lymphocytic and macrophage infiltrate, and a predominant macrophage pattern with osteolysis/aseptic loosening is to be classified as a type of ALTR. In research and clinical practice, definition of osteolysis should always be based on radiological images and comparative histopathological examination of the bone tissue. An evidence-based classification of ALTRs with emphasis on differential diagnosis should be developed for radiological diagnosis (MRI and ultrasound studies) through retrospective correlation with histopathological examination of the periprosthetic soft and bone tissue. With the ultimate aim of better patient care, the cooperation between clinical and basic researchers, stakeholders including patients, healthcare providers, financiers (governments and insurance companies), public health and regulatory agencies, industry, and the media (70) should be strengthened by conjoint postulation and fostering of a unified ALTRs-related terminology for the identification of patients and implants at risk and elucidation of the mechanisms of particle wear–host interaction . At last, a common effort by the medical and scientific community for the adoption of an evidence-based terminology for ALTRs to arthroplasty implants is even more urgent and necessary to avoid that ambiguous or incorrect diagnoses would become an integral part of the collection of digital real-world data used for their analysis by AI or machine learning techniques for evidence-based decision making.

## ICMJE Statement of Interest

The authors declare that there is no conflict of interest that could be perceived as prejudicing the impartiality of the work reported.

## Funding

The authors acknowledge the support for the article processing charge from the DFG (German Research Foundation, 393148499) and the Open Access Publication Fund of the University of Greifswald. JS acknowledges support by the Research Training Group ‘SYLOBIO’ (RTG 2901) funded by the DFG (German Research Foundation).

## References

[1] Vendittoli PA, Riviere C, Hirschmann MT, et al. Why personalized surgery is the future of hip and knee arthroplasty: a statement from the Personalized Arthroplasty Society. EFORT Open Rev 2023 8 874–882. (10.1530/eor-22-0096) 38038379 PMC10714387

[2] Morawietz L, Classen R, Schröder J, et al. Proposal for a histopathological consensus classification of the periprosthetic interface membrane. J Clin Pathol 2006 59 591–597. (10.1136/jcp.2005.027458) 16731601 PMC1860400

[3] Biological responses to metal implants. https://www.fda.gov/media/132446/download. Dated 2019. [press release].

[4] McGrory BJ, Jacobs JJ, Kwon YM, et al. Standardizing terms for tribocorrosion-associated adverse local tissue reaction in total hip arthroplasty. Arthroplast Today 2020 6 196–200. (10.1016/j.artd.2020.01.008) 32577461 PMC7303482

[5] Perino G, De Martino I, Zhang L, et al. The contribution of the histopathological examination to the diagnosis of adverse local tissue reactions in arthroplasty. EFORT Open Rev 2021 6 399–419. (10.1302/2058-5241.6.210013) 34267931 PMC8246109

[6] Hansen VJ, Greene ME, Bragdon MA, et al. Registries collecting level-I through IV data: institutional and multicenter use: AAOS exhibit selection. J Bone Jt Surg Am 2014 96 e160. (10.2106/jbjs.m.01458) 25232090

[7] Liebs TR, Splietker F & Hassenpflug J. Is a revision a revision? An analysis of national arthroplasty registries' definitions of revision. Clin Orthop Relat Res 2015 473 3421–3430. (10.1007/s11999-015-4255-4) 25791442 PMC4586197

[8] Morlock MM, Gomez-Barrena E, Wirtz DC, et al. Explant analysis and implant registries are both needed to further improve patient safety. EFORT Open Rev 2022 7 344–348. (10.1530/eor-22-0033) 35638602 PMC9257736

[9] Banerjee S, Cafri G, Isaacs AJ, et al. A distributed health data network analysis of survival outcomes: the International Consortium of Orthopaedic Registries perspective. J Bone Joint Surg Am 2014 96 (Supplement 1) 7–11. (10.2106/jbjs.n.00642) 25520413 PMC4271424

[10] Bohr A & Memarzadeh K. The rise of artificial intelligence in healthcare applications. In:. Artificial Intelligence in Healthcare 2020 25–60. (10.1016/B978-0-12-818438-7.00002-2)

[11] Li Z, Maimaiti Z, Fu J, et al. Global research landscape on artificial intelligence in arthroplasty: a bibliometric analysis. Digit Health 2023 9 20552076231184048. (10.1177/20552076231184048) 37361434 PMC10286212

[12] Sedrakyan A, Marinac-Dabic D, Campbell B, et al. Advancing the real-world evidence for medical devices through coordinated registry networks. BMJ Surg Interv Health Technol 2022 4 (Supplement 1) e000123. (10.1136/bmjsit-2021-000123) PMC9660584 36393894

[13] Pane J, Verhamme KMC, Villegas D, et al. Challenges associated with the safety signal detection process for medical devices. Med Devices 2021 14 43–57. (10.2147/mder.s278868) PMC7917351 33658868

[14] IMDRF. Adverse event terminology maintenance, 2020. (http://www.imdrf.org/workitems/wi-aet-maintenance.asp). Accessed on 12 June 2024.

[15] IMDRF. IMDRF terminologies for categorized adverse event reporting (AER): terms, terminology structure and codes, 2020. (http://www.imdrf.org/docs/imdrf/final/technical/imdrf-tech-200318-ae-terminologies-n43.pdf). Accessed on 12 June 2024.

[16] Campbell P, Mirra J & Catelas I. Histopathology of failed inter-Op acetabular components. Orthopaedic Proc 2004 86-B (Supplement IV) 427.

[17] Bonsignore LA, Goldberg VM & Greenfield EM. Machine oil inhibits the osseointegration of orthopaedic implants by impairing osteoblast attachment and spreading. J Orthop Res 2015 33 979–987. (10.1002/jor.22850) 25676177 PMC8201705

[18] Southern DA, Harrison JE, Romano PS, et al. The three-part model for coding causes and mechanisms of healthcare-related adverse events. BMC Med Inform Decis Mak 2021 21 376. (10.1186/s12911-022-01786-w) PMC8867615 35209889

[19] Krenn V, Morawietz L, Perino G, et al. Revised histopathological consensus classification of joint implant related pathology. Pathol Res Pract 2014 210 779–786. (10.1016/j.prp.2014.09.017) 25454771

[20] Perino G, Sunitsch S, Huber M, et al. Diagnostic guidelines for the histological particle algorithm in the periprosthetic neo-synovial tissue. BMC Clin Pathol 2018 18 7. (10.1186/s12907-018-0074-3) 30158837 PMC6109269

[21] Davis D & Morrison J. Hip arthroplasty pseudotumors: pathogenesis, imaging, and clinical decision making. J Clin Imaging Sci 2016 6 17. (10.4103/2156-7514.181493) 27195183 PMC4863402

[22] Davies A, Willert HG, Campbell P, et al. An unusual lymphocytic perivascular infiltration in tissues around contemporary metal-on-metal joint replacements. J Bone Jt Surg Am 2005 87 18–27. (10.2106/00004623-200501000-00005) 15634811

[23] Beaulé PE, Campbell P, Amstutz H, et al. Metallosis and metal-on-metal bearings. J Bone Joint Surg 2000 82 751–752. (10.2106/00004623-200005000-00022) 10819289

[24] Xia Z, Ricciardi B, Liu Z, et al. Nano-analyses of wear particles from metal-on-metal and non-metal-on-metal dual modular neck hip arthroplasty. Nanomedicine 2016 13 1205–1217. (10.1016/j.nano.2016.11.003) 27888094

[25] Schoon J, Hesse B, Rakow A, et al. Metal-specific biomaterial accumulation in human peri-implant bone and bone marrow. Adv Sci 2020 7 2000412. (10.1002/advs.202000412) PMC7578891 33101844

[26] Shah K, Pritt B & Alexander M. Histopathologic review of granulomatous inflammation. J Clin Tuberc Other Mycobact Dis 2017 7 1–12. (10.1016/j.jctube.2017.02.001) 31723695 PMC6850266

[27] Klopfleisch R & Jung F. The pathology of the foreign body reaction against biomaterials. J Biomed Mater Res 2017 105 927–940. (10.1002/jbm.a.35958) 27813288

[28] Perino G, Ricciardi BF, Jerabek SA, et al. Implant based differences in adverse local tissue reaction in failed total hip arthroplasties: a morphological and immunohistochemical study. BMC Clin Pathol 2014 14 39. (10.1186/1472-6890-14-39) 25242891 PMC4169255

[29] Fontenot AP, Falta MT, Kappler JW, et al. Beryllium-induced hypersensitivity: genetic susceptibility and neoantigen generation. J Immunol 2016 196 22–27. (10.4049/jimmunol.1502011) 26685315 PMC4685955

[30] Jacobs J, Urban R, Wall J, et al. Unusual foreign-body reaction to a failed total knee replacement: simulation of a sarcoma clinically and a sarcoid histologically. A case report. J Bone Jt Surg Am 1995 77 444–451. (10.2106/00004623-199503000-00015) 7890794

[31] Gallo J, Goodman S, Konttinen Y, et al. Particle disease: biologic mechanisms of periprosthetic osteolysis in total hip arthroplasty. Innate Immun 2013 19 213–224. (10.1177/1753425912451779) 22751380 PMC3712274

[32] Gallo J, Goodman S, Konttinen Y, et al. Osteolysis around total knee arthroplasty: a review of pathogenetic mechanisms. Acta Biomater 2013 9 8046–8058. (10.1016/j.actbio.2013.05.005) 23669623 PMC4003873

[33] Goodman S & Gallo J. Periprosthetic osteolysis: mechanisms, prevention and treatment. J Clin Med 2019 8 2091. (10.3390/jcm8122091) 31805704 PMC6947309

[34] Panez-Toro I, Heymann D, Gouin F, et al. Roles of inflammatory cell infiltrate in periprosthetic osteolysis. Front Immunol 2023 14 1310262. (10.3389/fimmu.2023.1310262) 38106424 PMC10722268

[35] Cordova L, Stresing V, Gobin B, et al. Orthopaedic implant failure: aseptic implant loosening – the contribution and future challenges of mouse models in translational research. Clin Sci 2014 127 277–293. (10.1042/cs20130338) 24827940

[36] Gogna P, Paladini P, Merolla G, et al. Metallosis in shoulder arthroplasty: an integrative review of literature. Musculoskelet Surg 2016 100 3–11. (10.1007/s12306-016-0408-1) 27900702

[37] Glazebrook M, Arsenault K & Dunbar M. Evidence-based classification of complications in total ankle arthroplasty. Foot Ankle Int 2009 30 945–949. (10.3113/fai.2009.0945) 19796587

[38] Mercer J, Penner M, Wing K, et al. Inconsistency in the reporting of adverse events in total ankle arthroplasty: a systematic review of the literature. Foot Ankle Int 2015 37 127–136. (10.1177/1071100715609719) 26445992

[39] Mahmoud K, Metikala S, O’Connor K, et al. Adverse events related to total ankle replacement devices: an analysis of reports to the United States Food and Drug Administration. Int Orthop 2021 45 2307–2312. (10.1007/s00264-021-04972-z) 33575857 PMC8494697

[40] Dalat F, Barnoud R, Fessy MH, et al. Histologic study of periprosthetic osteolytic lesions after AES total ankle replacement. A 22 case series. Orthop Traumatol Surg Res 2013 99 (Supplement 6) S285–S295. (10.1016/j.otsr.2013.07.009) 23978711

[41] van Wijngaarden R, van der Plaat L, Nieuwe Weme RA, et al. Etiopathogenesis of osteolytic cysts associated with total ankle arthroplasty, a histological study. Foot Ankle Surg 2015 21 132–136. (10.1016/j.fas.2015.02.004) 25937414

[42] Schipper ON, Haddad SL, Pytel P, et al. Histological analysis of early osteolysis in total ankle arthroplasty. Foot Ankle Int 2017 38 351–359. (10.1177/1071100716682333) 28367690

[43] Bracco P, Bellare A, Bistolfi A, et al. Ultra-high molecular weight polyethylene: influence of the chemical, physical and mechanical properties on the wear behavior. A review. Materials 2017 10 791. (10.3390/ma10070791) 28773153 PMC5551834

[44] Willert H-G & Semlitsch M. Reactions of the articular capsule to wear products of artificial joint prostheses. J Biomed Mater Res 1977 11 157–164. (10.1002/jbm.820110202) 140168

[45] Schmalzried TP, Kwong LM, Jasty M, et al. The mechanism of loosening of cemented acetabular components in total hip arthroplasty. Analysis of specimens retrieved at autopsy. Clin Orthop Relat Res 1992 274 60–78. (10.1097/00003086-199201000-00009) 1729024

[46] Goethem E, Poincloux R, Gauffre F, et al. Matrix architecture dictates three-dimensional migration modes of human macrophages: differential involvement of proteases and podosome-like structures. J Immunol 2009 184 1049–1061. (10.4049/jimmunol.0902223) 20018633

[47] Čermák V, Gandalovičová A, Merta L, et al. RNA-seq of macrophages of amoeboid or mesenchymal migratory phenotype due to specific structure of environment. Sci Data 2018 5 180198. (10.1038/sdata.2018.198) 30277482 PMC6167950

[48] Navratilova P, Emmer J, Tomas T, et al. Plastic response of macrophages to metal ions and nanoparticles in time mimicking metal implant body environment. Environ Sci Pollut Res Int 2024 31 4111–4129. (10.1007/s11356-023-31430-7) 38097843

[49] Di Laura A, Quinn PD, Panagiotopoulou VC, et al. The chemical form of metal species released from corroded taper junctions of hip implants: synchrotron analysis of patient tissue. Sci Rep 2017 7 10952. (10.1038/s41598-017-11225-w) 28887488 PMC5591307

[50] Xu J, Yang J, Nyga A, et al. Cobalt (II) ions and nanoparticles induce macrophage retention by ROS-mediated down-regulation of RhoA expression. Acta Biomater 2018 72 434–446. (10.1016/j.actbio.2018.03.054) 29649639 PMC5953279

[51] Jain N, Moeller J & Vogel V. Mechanobiology of macrophages: how physical factors coregulate macrophage plasticity and phagocytosis. Annu Rev Biomed Eng 2019 21 267–297. (10.1146/annurev-bioeng-062117-121224) 31167103

[52] Ort M, Geissler S, Rakow A, et al. The allergic bone marrow? The immuno-capacity of the human bone marrow in context of metal-associated hypersensitivity reactions. Front Immunol 2019 10 2232. (10.3389/fimmu.2019.02232) 31620137 PMC6759684

[53] Steinbeck MJ, Jablonowski LJ, Parvizi J, et al. The role of oxidative stress in aseptic loosening of total hip arthroplasties. J Arthroplast 2014 29 843–849. (10.1016/j.arth.2013.09.001) PMC3965616 24290740

[54] Mesko JW, D'Antonio JA, Capello WN, et al. Ceramic-on-Ceramic hip outcome at a 5- to 10-year interval: has it lived up to its expectations? J Arthroplast 2011 26 172–177. (10.1016/j.arth.2010.04.029) 20580193

[55] Kim K, Zhu M, Cavadino A, et al. Failed debridement and implant retention does not compromise the success of subsequent staged revision in infected total knee arthroplasty. J Arthroplast 2019 34 1214–1220.e1. (10.1016/j.arth.2019.01.066) 30826164

[56] Kim Y-H, Park J-W & Kim J-S. Alumina delta-on-alumina delta bearing in cementless total hip arthroplasty in patients aged <50 years. J Arthroplast 2016 31 2209–2214. (10.1016/j.arth.2016.03.016) 27067468

[57] Yoon TR, Rowe SM, Jung ST, et al. Osteolysis in association with a total hip arthroplasty with ceramic bearing surfaces. J Bone Joint Surg 1998 80 1459–1467. (10.2106/00004623-199810000-00007) 9801214

[58] Urban R, Jacobs J, Tomlinson M, et al. Dissemination of wear particles to the liver, spleen, and abdominal lymph nodes of patients with hip or knee replacement. J Bone Jt Surg Am 2000 82 457–477. (10.2106/00004623-200004000-00002) 10761937

[59] Hall D, Pourzal R, Jacobs J, et al. Metal wear particles in hematopoietic marrow of the axial skeleton in patients with prior revision for mechanical failure of a hip or knee arthroplasty. J Biomed Mater Res B Appl Biomater 2018 107 1930–1936. (10.1002/jbm.b.34285) 30501001 PMC6542727

[60] Hayter CL, Gold SL, Koff MF, et al. MRI findings in painful metal-on-metal hip arthroplasty. Am J Roentgenol 2012 199 884–893. (10.2214/ajr.11.8203) 22997383

[61] Nawabi D, Hayter C, Su E, et al. Magnetic resonance imaging findings in symptomatic versus asymptomatic subjects following metal-on-metal hip resurfacing arthroplasty. J Bone Jt Surg Am Vol 2013 95 895–902. (10.2106/jbjs.k.01476) 23677356

[62] Koff MF, Burge AJ, Koch KM, et al. Imaging near orthopedic hardware. J Magn Reson Imag 2017 46 24–39. (10.1002/jmri.25577) PMC5464983 28152257

[63] Petscavage-Thomas JM & Ha A. Best practices: best imaging modality for surveillance of metal-on-metal hip arthroplasty. Am J Roentgenol 2021 216 311–317. (10.2214/ajr.19.22344) 33325734

[64] Saadi SB, kazemi RRO, Amirabadi A, et al. Osteolysis: a literature review of basic science and potential computer-based image processing detection methods. Intell Neurosci 2021 2021 21. (10.1155/2021/4196241) PMC8505126 34646317

[65] Ricciardi B, Nocon A, Jerabek S, et al. Histopathological characterization of corrosion product associated adverse local tissue reaction in hip implants: a study of 285 cases. BMC Clin Pathol 2016 16 3. (10.1186/s12907-016-0025-9) 26924942 PMC4769839

[66] Lainiala OS, Reito AP, Nieminen JJ, et al. Declining revision burden of metal-on-metal hip arthroplasties. J Arthroplast 2019 34 2058–2064.e1. (10.1016/j.arth.2019.04.058) 31174908

[67] Laaksonen I, Galea V, Connelly J, et al. Progression of adverse local tissue reaction in ASR metal-on-metal hip arthroplasty: a longitudinal MARS-MRI study at mid- to long-term. Hip Int 2019 31 369–377. (10.1177/1120700019894668) 31868016

[68] Schulze F, Perino G, Rakow A, et al. Noninfectious tissue interactions at periprosthetic interfaces. Die Orthopädie 2023 52 186–195. (10.1007/s00132-023-04352-y) 36853395

[69] Bozzato L, Ferrari M & Trombetta A. Building a domain ontology from glossaries: a general methodology. Proceedings of the 5th Workshop on Semantic Web Applications and Perspectives 2008 426.

[70] Lübbeke A, Carr AJ & Hoffmeyer P. Registry stakeholders. EFORT Open Rev 2019 4 330–336. (10.1302/2058-5241.4.180077) 31210971 PMC6549107

